# Chronic substance use and self-harm in a primary health care setting

**DOI:** 10.4102/phcfm.v10i1.1544

**Published:** 2018-06-19

**Authors:** Elsie Breet, Jason Bantjes, Ian Lewis

**Affiliations:** 1 Department of Psychology, Stellenbosch University, South Africa; 2 Department of Psychiatry, University of Cape Town, South Africa

## Abstract

**Background:**

Chronic substance use (CSU) is associated with health problems, including self-harm, placing a significant burden on health care resources and emergency departments (EDs). This is problematic in low- and middle-income countries like South Africa (SA), where primary care facilitates and emergency departments (EDs) are often poorly resourced.

**Aim:**

To investigate the epidemiology of CSU and self-harm and to consider the implications for primary health care service delivery and suicide prevention in SA.

**Methods:**

Data were collected from 238 consecutive self-harm patients treated at the emergency department (ED) of an urban hospital in SA. The data were analysed using bivariate and multivariate analyses.

**Results:**

Approximately 37% of self-harm patients reported CSU. The patients in the CSU subgroup, compared to other self-harm patients, were more likely to be men (odds ratio [OR] = 8.33, 95% confidence interval [CI] = 3.19–20.9, *p* < 0.001), to have self-harmed by inflicting damage to their body tissue OR = 4.45, 95% CI = 1.77–11.2, *p* < 0.01) and to have a history of self-harm (OR = 3.71, 95% CI = 1.44–9.54, *p* = 0.007). A significantly smaller proportion of CSU patients, compared to other self-harm patients, were referred for psychiatric assessment (OR = 8.05, 95% CI = 4.16–15.7, *p* < 0.001).

**Conclusion:**

The findings of this study confirm that CSU is associated with greater service utilisation and repetition of self-harm among patients in primary health care settings. Treating self-harm as the presenting problem within primary care settings does not necessarily ensure that patients receive the care that they need. It might be helpful to include psychiatric assessments and screening for CSU as an integral component of care for self-harm patients who present in primary health care settings.

## Introduction

Chronic substance use (CSU) is understood as the recurrent and harmful use of substances over an extended period of time; this includes both substance use disorders and recurring patterns of hazardous substance use.^1,2,3,4^ CSU is associated with adverse health outcomes among people who visit primary health care settings.^5,6^ For example, CSU is associated with serious medical health issues (e.g. cardiac problems) and mental health issues (e.g. mood disturbances) that complicate treatment of acute health problems.^7^ CSU also contributes to interpersonal violence and other forms of injury that require emergency medical care.^8^ Likewise, it is well established that CSU increases the risk for self-harm among patients attending primary health care services.^9,10,11^ CSU, especially when co-occurring with other medical or mental health issues, increases the use of high-cost health services.^12^ A recent systematic review of associations between substance use and suicidal behaviour in low-and middle-income countries (LMICs) showed that acute substance use and CSU were associated with suicidal ideation and behaviour; highlighting the importance on ensuring that assessment of CSU and interventions that reduce the harmful use of substances should be integral to suicide prevention.^13^ This is particularly important in LMICs, like South Africa (SA), where primary care facilities are often poorly resourced, understaffed, overcrowded and open for limited hours.^14,15^ No studies have explored the epidemiology of CSU and self-harm, or the implications for primary health care in SA. Knowledge of the epidemiology of CSU and self-harm in primary health care settings and emergency departments (EDs) has the potential to contribute to improved service delivery and suicide prevention in primary health care settings. It is within this context that we set out to explore the epidemiology of CSU and self-harm among patients presenting to an ED at an urban hospital in SA.

The term self-harm was defined as an intentional non-fatal self-injury or self-poisoning regardless of the level of intent to die.^16,17^ The term includes deliberate self-harm and suicide attempts but does not include other forms of self-injurious behaviours that are habitual (i.e. non-suicidal self-injury). The reason for using this broad definition of self-harm is that expert consensus suggests that suicide prevention should focus on the full range of self-injurious behaviours irrespective of the level of intent to die.^18^ The definition of self-harm used in this study is also congruent with the definition used by the World Health Organization (WHO) to describe non-fatal suicidal behaviour, as well as other studies that have investigated self-harm.^16,17,19^

CSU has been consistently associated with self-harm, although prevalence rates seem to vary by geographic region and gender. A study 3 567 084 suicide attempt-related visits to an ED in the United States showed that 12.1% of self-harm patients met diagnostic criteria for substance-related disorders.^9^ A longitudinal study among 1943 self-harm patients in England reported alcohol misuse among 36.1% of self-harm patients.^20^ Gender accounts for some variability in reported prevalence of CSU among people who self-harm.^21^ CSU is generally higher among men who self-harm compared to women, though some evidence shows this phenomenon is increasing among women.^22^ Among 400 Iranian patients who had made suicide attempts, 15.8% reported a history of drug abuse.^23^ A study among 131 patients attending public primary care in SA found that patients had made suicide attempts were 3 times more likely to also report harmful alcohol use even when controlling for demographic characteristics (OR = 3.01, CI = 1.83–4.95, *p* < 0.001).^24^

Research in high-income countries has consistently demonstrated that CSU is a risk factor for repetition of self-harm,^25^ though evidence suggests that other factors interact with CSU to increase the risk of repetition. For example, a population-based study from Western Australia (*N* = 16 966) reported that 6 % of adolescents and 8 % of young adults engaged in repetition of self-harm within 7 days of an index incident of self-harm and that substance use disorders were significantly associated with risk of repetition (OR = 1.76, 95% CI = 1.46–2.13, *p* < 0.01).^26^ This same study highlighted that other significant risk factors for repetition of self-harm were impulse-control disorders and personality disorders.^26^ Data from another population-based study in Canada (*N* = 81 675) showed that in addition to alcohol dependence, other factors such as symptoms of depression, lower socio-economic status (SES) and younger age predicted repetition of self-harm.^25^ These data highlight the importance of a thorough psychiatric assessment, which includes screening for substance use and considering the interaction of variables such as age, gender, SES and comorbid psychiatric disorders, among self-harm patients who present for treatment in primary health care settings.

Self-harm patients account for a significant proportion of those who present for treatment in the ED. In England, there are an estimated 200 000 admissions to EDs per year for self-harm.^27^ Data from the WHO multi-site intervention study on suicidal behaviour reported that in an SA setting, 47% of suicide attempters sought medical attention in an ED.^28^

In high-income countries, self-poisoning is consistently the most common method of self-harm among patients treated in EDs, although damage to body tissue (e.g. cutting of the body tissue or hanging) is also common.^29,30^ Similarly, self-poisoning (e.g. pesticide poisoning) is the leading method of self-harm in LMICs.^10,31^ Research has consistently shown associations between substance intoxication and method of self-harm,^2^ though research in this area on the association of CSU with method of self-harm is scant.

For some patients, a visit to the ED presents an opportunity not only to receive treatment or referral for the presenting problem (i.e. self-harm) but also to gain access to knowledge and resources to address the underlying predisposing and precipitating factors contributing to the presenting problem (e.g. CSU).^10^ Bergen and colleagues demonstrated that receiving a psychiatric assessment accounts for a 13% (95% CI = 1% – 24%) decrease in risk of repetition of self-harm.^22^ Therefore, a visit to the ED that includes a psychiatric assessment could decrease the risk of repetition of self-harm and ensure that patients receive the help that they need by, for example, providing an opportunity to reinforce health-promoting behaviours, providing psychoeducation and making appropriate referrals.^32^ Despite the benefit of a psychiatric assessment in reducing self-harm, such assessments are not routinely conducted before discharge from the ED.^33^ In SA, early detection of risk for self-harm and necessary care is not always feasible or available to everyone, as limited skills and resources hinder adequate and timeous treatment or referral.^34^ The shortage of mental health care professionals in South African primary health care settings and the reality that primary health care is still primarily biomedical in its orientation mean that it is not always possible to provide psychiatric assessments or psychosocial interventions in these settings, even when they are indicated.^35,36^

Though substance use is a growing public health problem in LMICs,^37^ there are no published data on the risk for self-harm among those with CSU in SA. A better understanding of the epidemiology of CSU and self-harm is a first step in better understanding how to organise care for these patients, provide early detection and deliver effective interventions for this vulnerable population. The aim of this study was to compare the two groups of self-harm patients, those with and those without CSU, with respect to their demographic characteristics, method of self-harm, suicidal intent, history of self-harm, referral for hospital admission, and whether or not a referral for a psychiatric assessment was received.

## Methods

### Study design, setting and sampling

Data were collected for this cohort study from 270 consecutive self-harm patients presenting to the ED of a hospital in SA between 16 June 2014 and 29 March 2015. The hospital is a large public hospital in an urban city with a catchment area of 1.5 million people.^38^

Though data were collected for 270 patients, we included 238 patients in the final statistical analysis. Patients were excluded if their files were missing or if there was not sufficient information available in the patient file (17 patients), if the patient had already been included in the sample on a prior presentation to the hospital during the period of data collection (9 patients), if the patient left the hospital before their information was captured (1 patient), or where patients died as a result of their injuries (5 patients).

### Measures

Data were collected from patient records that contained information recorded by doctors in the ED. The recording of these data is part of the routine clerking of all self-harm patients treated in the ED. These data were extracted from patient records with the help of an experienced psychiatric nurse. Quality checks for possible errors and missing data were performed throughout data collection. The following data were collected:

#### Demographic information

Patients’ gender, age, race, relationship status, whether or not they had dependents, completed level of education and employment status were recorded. Socio-economic status was also recorded as low to moderate SES (0 ZAR to 76 800 ZAR) and high SES (76 801 ZAR to 2 547 601 ZAR) based on annual family income. At the time of this study, 15.72 ZAR = 1 US dollar.

#### Time and day of presentation at the emergency department

Time and day of admission was recorded as part of routine clerking of all patients treated in the ED.

#### Chronic substance use

Self-reports of a history of substance use disorder, and recurrent or habitual harmful use of alcohol or illicit drugs were recorded.^1,2,3,4^ There is evidence, internationally and in the South African context, to show that self-report measures of substance use are valid and reliable. Self-report of alcohol, cannabis and methamphetamine use are consistent with measures of substance use biomarkers.^39,40^

#### Clinical features of self-harm

Method(s) of self-harm, whether or not the incident was impulsive and whether or not the patient was referred for a psychiatric assessment. The level of admission was recorded as: (1) treated in ED and discharged or, (2) transferred to secondary or tertiary level care (intensive care unit [ICU], high care, medical or surgical ward, or emergency psychiatric unit).

#### Level of suicidal intent

The 12-item Pierce Suicidal Intent Scale (PSIS) was used to measure suicidal intent among patients.^41^ The PSIS scores range from 0 to 25, where scores between 0 and 11 indicate low to moderate suicidal intent, whilst scores higher than 11 indicate severe suicidal intent.

#### History of self-harm

Patients’ self-report of the number of previous episodes of self-harm was recorded.

### Data analysis

Data were captured, cleaned and analysed with Statistical Package for the Social Sciences’ (SPSS v.19) (SPSS Inc., Chicago, IL, USA). Three quality control checks were completed before the data were analysed. Descriptive statistics were calculated to provide an overview of the data. The association between CSU and self-harm was calculated using chi-square statistics or Fisher’s exact test for categorical variables. Odds ratios and 95% confidence intervals were calculated where appropriate. Between-group analysis of the continuous variables age and length of stay in the hospital were calculated using the Mann–Whitney test for non-normal distributions. Logistic regression analysis was used to determine the relationship between CSU and gender, age, SES, having dependents or not, history of self-harm, impulsive self-harm, method of self-harm, level of suicidal intent, psychiatric referral, and referral for secondary or tertiary care. Statistical significance was considered at *p* < 0.05.

### Ethical considerations

Ethical approval to conduct the study was granted by the Health Sciences Research Ethics committee of Stellenbosch University (reference number N13/05/074) and the University of Cape Town (reference number 645/2013). We obtained institutional permission from the hospital before patient records were accessed. The information collected from each patient record was assigned a unique number and stored on a password protected computer to protect patient confidentiality.

## Results

### Demographic characteristics of sample

A total of 238 patients were included in this study of which 37% reported a history of CSU. Table 1 presents the sample characteristics of the CSU patients and other self-harm patients. The mean age for the subgroup of CSU patients was 32.4 (12.5 SD) years, and the age range was 13–82 years. Most of the patients in the subgroup of CSU patients were mixed race^1^ (41.6%), not in a relationship (87.6%), unemployed (76.4%), from a low to moderate SES (52.8%), had completed a Grade 12 school level of education (46.1%) and did not have dependents (71.9%). The subgroup of CSU patients were more likely to be men when compared to the other self-harm patients (χ^2^ = 24.4, *df* = 1, *p* < 0.001, OR = 3.93, 95% CI = 2.18–7.13). In the logistic regression, men were approximately eight times more likely to report CSU when controlling for demographic variables, and impulsive self-harm or a history of self-harm (OR = 8. 8.33, 95% CI = 3.19 – 20.9, *p* < 0.001) (see Table 2).

**TABLE 1:** Description and comparison of sample demographic characteristics by chronic substance use (*N* = 238).

Variable	Yes† (*n* = 89)		No‡ (*n* = 149)		χ^2^	*df*	*p*	OR	CI
	*n*	%	*n*	%					
**Gender**	-	-	-	-	24.4	1	0.000	3.93	2.18–7.13
Male	54	60.7	42	28.2	-	-	-	-	-
Female	35	39.3	107	71.8	-	-	-	-	-
Mean (SD) Age (years)	32.4	12.5	31.0	14.6	-	-	-	-	-
**Race§**	-	-	-	-	3.07	3	0.380	-	-
Black people	27	30.3	55	36.9	-	-	-	-	-
Asian people or Moslem people	4	4.5	4	2.7	-	-	-	-	-
Mixed race people ¶	37	41.6	66	44.3	-	-	-	-	-
White people	16	18	17	11.4	-	-	-	-	-
Not known	5	5.6	7	4.7	-	-	-	-	-
**Relationship status**	-	-	-	-	3.72	1	0.054	-	-
Married or in a relationship	11	12.3	33	22.1	-	-	-	-	-
Not in a relationship	78	87.6	114	77.2	-	-	-	-	-
Not Known	0	-	1	0.7	-	-	-	-	-
**Have dependents**	-	-	-	-	3.56	1	0.059	-	-
No dependents or pregnant	64	71.9	91	61.1	-	-	-	-	-
Dependents	23	25.8	57	38.3	-	-	-	-	-
Not known	2	2.2	1	0.7	-	-	-	-	-
**Completed level of education**††	-		-	-	1.45	2	0.484	-	-
Primary school	33	37.1	67	45	-	-	-	-	-
Secondary school	41	46.1	59	39.6	-	-	-	-	-
Tertiary school (undergraduate or postgraduate)	15	16.9	23	15.4	-	-	-	-	-
**Employment status‡‡**	-	-	-	-	0.000	1	0.989	-	-
Employed	19	21.3	32	21.5	-	-	-	-	-
Unemployed (unemployed, scholar, student)	68	76.4	114	76.6	-	-	-	-	-
Not known	2	2.2	3	2	-	-	-	-	-
**SES§§ (*n* = 216)**	-	-	-	-	0.615	1	0.433	-	-
Low to moderate SES (ZAR0 to ZAR76 800)	47	52.8	84	56.4	-	-	-	-	-
High SES (ZAR76 801 to ZAR2 547 601)	35	39.3	50	33.6	-	-	-	-	-
Not known	7	7.9	15	10.1	-	-	-	-	-

Notes: Chi-square statistics were calculated for categorical variables: gender, race, having dependents or no dependents, completed level of education, and SES. Mann–Whitney U test was used for between-group analyses of continuous variables with non-normal distribution: Mean age (years).

SES, socio-economic status; OR, Odds Ratio; CI, confidence interval; SD, standard deviation.

† , *n* = 89 patients with chronic substance use;

‡ , *n* = 149 other self-harm patients;

§ , The term ‘race’ may be offensive in some countries; however, this is an official term used in South Africa.

¶ , The term ‘mixed race’ may be offensive in some countries; however, this is an official term used in South Africa;

†† , Primary school is from 1st grade to 7th grade in the United States; Secondary school is from the 8th grade to 12th grade (senior in the United States); Tertiary school is any diploma or university degree after completing Grade 12;

‡‡ , Employment status of the six participants who indicated that they were retired were included in the employed category as they qualify to receive old age pension from the state worth R1420 per month;

§§ , R15.72 = 1 $US.

**TABLE 2:** Binary logistical regression analysis: Summary of predictors in each model.

Model	Predictor	Outcome	*β*	s.e.	Wald χ^2^	*p*	OR	CI
1	Gender	CSU	2.12	0.470	20.4	0.000	8.33	3.19–20.9
	Age	-	-0.009	0.018	0.216	0.642	0.991	0.956–1.03
	SES	-	0.443	0.461	0.922	0.337	1.56	0.630–3.85
	Dependents	-	0.467	0.498	0.880	0.348	1.60	0.601–4.23
	History of self-harm	-	1.31	0.482	0.739	0.007	3.71	1.44–9.54
	Impulsive self-harm	-	1.07	0.519	4.27	0.039	2.92	1.06–8.08
2	CSU	Self-poison versus damage to bodily tissue	1.49	0.470	10.1	0.001	4.45	1.77–11.2
3	CSU	PSIS†	-0.126	0.577	0.047	0.828	0.882	0.285–2.73
4	CSU	Psychiatric referral	0.133	0.508	0.068	0.794	1.14	0.422–3.09
5	CSU	Impulsive act	-0.711	0.390	3.32	0.068	0.491	0.229–1.06
6	CSU	ED and discharge versus long stay medical ward	-0.171	0.311	0.301	0.583	0.843	0.458–1.55

Notes: Model 1: Gender, age, SES, having dependents, previous self-harm and impulsive self-harm predict CSU. Model 2: CSU predict the method of self-harm, while controlling for gender, age and SES. Model 3: CSU predict suicidal intent, while controlling for gender, age, previous self-harm and SES. Model 4: CSU predict referral for psychiatric assessment, while controlling for gender, age, SES and suicidal intent. Model 5: CSU predict impulsive self-harm, while controlling for gender, age and SES. Model 6: CSU predict being treated in the ED and discharged or referral to hospital, while controlling for gender, age and SES.

OR, odds ratio; CI, confidence interval; CSU, chronic substance use; SES, socio-economic status; ED, emergency department; PSIS, Pierce Suicidal Intent Scale; s.e. standard error; *β*, beta.

† , Low to moderate suicide intent = a PSIS of 11 or lower; high suicide intent = PSIS score of 12 or more.

### Methods of self-harm

The prevalence of different methods for self-harm and the comparison between the subgroup of CSU patients and other self-harm patients is presented in Table 3. Most of the subgroup of CSU patients used self-poisoning (64%) as the method of self-harm. Among the patients who utilised self-poisoning, abuse of prescription medication (36%) was the most common method of self-harm. A significantly greater proportion of the subgroup of CSU patients, compared to other self-harm patients (26.8% vs. 7.4%, *p* < 0.001), utilised damage to body tissue as their method of self-harm (χ^2^ = 16.8, *df* = 1, *p* < 0.001, OR = 4.60, 95% CI = 2.01–10.7). In the logistic regression analysis, the CSU self-harm patients, compared to other self-harm patients, were five times more likely to use damage to body tissue as the method of self-harm (OR = 4.45, 95% CI = 1.77–11.2, *p* < 0.001) (see Table 2).

**TABLE 3:** Description and comparison of clinical features by CSU.

Variable	Yes (*n* = 89)		No (*n* = 149)		χ^2^	*df*	*p*	OR	CI
	*n*	%	*n*	%					
**Method of self-harm**	-	-	-	-	-	-	-	-	-
Self-poison	57	64.0	136	91.3	26.9	1	0.000	5.87	2.73–12.8
Prescription medication	32	36.0	66	44.3	-	-	-	-	-
Non-prescription medication	10	11.2	23	15.4	-	-	-	-	-
Ingestion or inhalation of poison	5	5.6	14	9.4	-	-	-	-	-
Mixed method of self-poisoning	10	11.2	33	24.3	-	-	-	-	-
Damage body tissue	24	26.8	11	7.4	16.8	1	0.000	4.60	2.01–10.7
Laceration	9	10.1	6	4.0	-	-	-	-	-
Hanging	9	10.1	3	2	-	-	-	-	-
Asphyxiation	1	1.1	0	-	-	-	-	-	-
Immolation	1	1.1	0	-	-	-	-	-	-
Jumped off a height	2	2.2	0	-	-	-	-	-	-
Jumped in front of a train	2	2.2	2	1.3	-	-	-	-	-
Mixed method (i.e. self-poison and damage to body tissue)	6	6.7	2	1.3	-	-	-	-	-
Not known	2	2.2	0	-	-	-	-	-	-
**Pierce Suicide Intent Scale (PSIS)†**	-	-	-	-	0.037	1	0.847	-	-
Low to moderate suicide intent	26	29.2	60	40.3	-	-	-	-	-
High suicide intent	12	13.5	30	20.1	-	-	-	-	-
Not known	51	57.3	59	39.6	-	-	-	-	-
**Received a psychiatric assessment**	-	-	-	-	49.0	1	0.000	8.05	4.16–15.7
Yes	18	20.2	100	67.1	-	-	-	-	-
No	71	79.8	49	32.9	-	-	-	-	-
**History self-harm**	-	-	-	-	7.47	1	0.006	2.51	1.22–5.16
History of self-harm	45	50.6	44	29.5	-	-	-	-	-
No history of self-harm	20	22.5	49	32.9	-	-	-	-	-
Not known	24	27	56	37.6	-	-	-	-	-
**Impulsive self-harm**	-	-	-	-	8.15	1	0.004	-	-
Yes	13	14.6	43	28.9	-	-	-	-	-
No	76	85.4	94	63.8	-	-	-	-	-
Not known	6	6.7	11	7.4	-	-	-	-	-

Notes: Total sample = 238. Chi-square statistics were calculated for categorical variables: method of self-harm, level of suicidal intent, and whether or not a psychiatric assessment was received, previous attempt of self-harm, and impulsive act.

OR, odds ratio; CI, confidence interval.

† , Low to moderate suicide intent = a PSIS of 11 or lower; high suicide intent = PSIS score of 12 or more.

### Level of suicidal intent

A slightly smaller proportion of CSU patients, when compared to other self-harm patients, reported low to moderate suicidal intent (29.2% vs. 40.3%, *n* = 26 vs. 60, *p* = 0.847). Likewise, only 13.5% of CSU patients reported high suicidal intent, whereas 20.1% of other self-harm patients reported high suicidal intent (Table 3). More than half (57.3%) of the CSU patients did not receive a suicidal intent assessment, even though this is part of the routine care of self-harm patients in this setting. In the chi-square analysis, there was no significant difference in the level of suicidal intent between the subgroup of CSU patients and other self-harm patients. Likewise, in the logistic regression analysis, CSU did not predict suicidal intent (see Table 2).

### Referral for psychiatric assessment

Only 20.2% of CSU self-harm patients were referred for a psychiatric assessment (see Table 3), compared to 67.1% of other self-harm patients. In the chi-square analysis, the subgroup of CSU patients were eight times less likely to receive a psychiatric assessment compared to other self-harm patients (χ^2^ = 49, *df* = 1, *p* < 0.001, OR = 8.05, 95% CI = 4.16–15.7). In the logistic regression analysis, this association did not remain significant, when controlling for gender, age, SES and suicidal intent (see Table 2).

### History of self-harm

Half (50.6%) of the CSU self-harm patients reported a history of self-harm (Table 3), compared to 29.5% of other self-harm patients (50.6% vs. 29.5%, *p* = 0.006). In the chi-square analysis, CSU was significantly associated with a history of self-harm (χ^2^ = 2.51, *df* = 1, *p* = 0.006). Results from the logistic regression showed that patients who had a history of self-harm were approximately 4 times more likely to be in the CSU subgroup, when controlling for gender, age, SES, having dependents and impulsive self-harm (OR = 3.71, 95% CI = 1.44–9.54, *p* = 0.007) (see Table 2).

### Impulsive self-harm

A significantly smaller proportion of CSU patients, when compared to other self-harm patients, reported that their self-harm was impulsive (14.6% vs. 28.9%, *p* = 0.004) (Table 3). Results from the chi-square analysis showed that an impulsive act of self-harm was significantly associated with CSU (χ^2^ = 2, *df* = 2, *p* = 0.004). In the logistic regression analysis, this association did not remain significant when controlling for gender, age and SES (see Table 2).

### Time and day of presentation to the emergency department

A slightly larger proportion of CSU patients, when compared to other self-harm patients, presented to the ED between 07:00 and 17:00 during the day (44.9% vs. 42.3%, *p* = 0.651) (Table 4). For both CSU patients and other self-harm patients, the largest proportion of patients presented to the ED after hours or during the night shift (i.e. between 17:00 and 07:00) (51.7% vs. 55%, *p* = 0.651). There were no significant differences between the CSU patients and other self-harm patients with regard to time of presentation to the ED.

**TABLE 4:** Details of admission required by CSU (*N* = 238).

Variable	Yes† (*n* = 89)		No‡ (*n* = 149)		χ^2^	*df*	*p*	OR	CI
	*n*	%	*n*	%					
**Time of presentation to ED**	-	-	-	-	0.205	1	0.651	-	-
Day shift (07:00 to 17:00)	40	44.9	63	42.3	-	-	-	-	-
Night shift or after hours	46	51.7	82	55	-	-	-	-	-
Not known	3	3.4	4	2.7	-	-	-	-	-
**Day of presentation to ED**	-	-	-	-	1.589	1	0.207	-	-
Weekday	64	71.9	94	63.1	-	-	-	-	-
Weekend day	25	28.1	53	35.6	-	-	-	-	-
Not known	0	-	2	1.3	-	-	-	-	-
**Referral for hospital admission**									
Treated in casualty and discharged	31	34.8	53	35.8	0.023	1	0.890	1.04	0.580–1.88
Admitted to short stay medical unit	21	23.6	43	29.1	0.840	1	0.359	1.326	0.695–2.540
ICU or high care	6	6.7	11	7.5	0.040	1	0.842	1.11	0.362–3.52
Admitted to long-stay medical or surgical ward	11	12.4	6	4.1	5.76	1	0.016	3.34	1.09–10.6
Admitted to emergency psychiatric unit	34	38.2	56	37.6	0.009	1	0.924	1.027	0.576–1.83

Note: Chi-square statistics were calculated for categorical variables: treated in casualty and discharged; admitted to short stay medical unit; ICU or high care; admitted to long-stay medical or surgical ward; admitted to emergency psychiatric unit; transferred to tertiary psychiatric hospital. Mann-Whitney *U* test was used for between-group analyses of continuous variables with non-normal distribution: Mean number of days spent in each unit.

ED, emergency department; OR, odds ratio; CI, confidence interval.

† , *n* = 89 patients with CSU;

‡ , *n* = 149 of other self-harm patients.

For both the CSU patients and other self-harm patients, the greatest proportion of patients presented to the ED on a weekday (71.9% vs. 63.1%, *p* = 0.207). A smaller proportion of CSU patients, when compared to other self-harm patients, presented to the ED on a Saturday or Sunday (28.1% vs. 35.6%, *p* = 0.207).

### Referral for hospital admission

Details about the number of self-harm patients referred for inpatient treatment are presented in Table 2. A slightly smaller proportion of CSU self-harm patients, compared to other self-harm patients, were treated in the ED and discharged (34.8% vs. 35.8%, *p* = 0.890), referred to a short-stay medical unit (23.6% vs. 29.1%, *p* = 0.359) and referred to Intensive Care Unit (ICU) or high care (6.7% vs. 7.5%, *p* = 0.842). CSU was significantly associated with a referral to a long-stay medical or surgical ward (χ^2^ = X = 5.76, *df* = 1, p = 0.016, OR = 3.34 95% CI = 1.09–10.6). This finding did not remain significant in the logistic regression analysis, when controlling for gender, age, SES and suicidal intent (see Table 2).

## Discussion

More than a third of the self-harm patients presenting at the ED of an urban hospital in SA reported CSU. This finding is similar to results reported in studies from high-income countries^20^ and other sub-Saharan African countries.^42^ Findings from other LMICs correspond with our finding that a greater proportion of men, compared to women, who self-harm also report CSU.^43^ Our data are also consistent with international studies showing that CSU is associated with repetition of self-harm.^22^ Our findings further support the epidemiological data that CSU is associated with greater medical service utilisation from primary health care settings,^44^ which highlights the need for psychiatric services to be an integral component of primary care.^12^

Our findings are congruent with other studies that report self-poisoning as a leading method of self-harm.^29,30,31^ Despite high rates of self-poisoning as the method of self-harm among both groups, a statistically significant greater proportion of the subgroup of CSU patients used damage to body tissue as their method of self-harm. This is a noteworthy finding given that damage to body tissue (e.g. hanging or cutting of body tissue) is typically more lethal,^32^ and causes severe injury requiring hospital-based care and longer hospital admissions.^45^

Patients with a history of self-harm were approximately four times more likely to be in the subgroup of CSU patients, when compared to other self-harm patients. There is an abundance of literature showing strong associations between CSU and repetition of self-harm.^25,46,47^ Furthermore, our results showed that the association between CSU and self-harm was not influenced by gender, age, SES, having dependents or not, and impulsive self-harm. However, the available literature suggests that other factors such as impulse-control disorders, personality disorders and symptoms of depression contribute to the increased risk for repetition of self-harm among people with CSU.^25,26^ A psychiatric assessment among CSU self-harm patients treated in primary health care settings is one potentially useful way of identifying other factors such as comorbid psychopathology that increase the risk for repetition of self-harm.

The finding that not all patients who present to the ED following self-harm receive a psychiatric assessment or referral is consistent with international practices.^30^ Likewise, a smaller proportion of CSU patients, compared to other patients, received a psychiatric assessment. The lack of psychiatric assessment is worrying given that a greater proportion of CSU patients compared to other self-harm patients reported a history of self-harm. The lack of a psychiatric assessment is a lost opportunity for intervention or for putting these patients in contact with substance abuse treatment facilities such as arranging referrals to specialist alcohol and drug treatment services. Integrating a psychiatric assessment within primary care could be an important component of preventing repetition of self-harm by ensuring that adequate treatment strategies are followed; psychiatric assessments also provide an opportunity to refer self-harm patients to available mental health services.^31,48^

Psychiatric assessment or screening to detect CSU among self-harm patients within primary health care will need to be planned carefully in SA to ensure that patients receive the care that they need without further exacerbating the burden on the already strained health care system.^34^ Health care providers may be disinclined to screen for CSU among self-harm patients when adequate treatment or referral resources are limited or not available. A study among substance-using patients attending public primary care services proposes that future research is required to investigate effective ways of improving resources that address substance use among primary health care patients presenting for suicidal behaviour.^49^

In LMICs, people with mental health problems do not typically have access to specialised health care services and therefore frequently seek help in primary health care settings, such as EDs.^50^ Larkin and Beautris argue that providing psychiatric services in the ED is vital in suicide prevention as patients often do not attend or adhere to treatment post-discharge.^51^ These studies highlight the need to utilise ED visits as a vital opportunity to provide necessary care for self-harm patients with CSU. Though psychiatric services are generally thought of as specialised services (i.e. secondary or tertiary care), they also have a place as a subspecialty in primary health care within low-resource settings.^12^ This is particularly important given that poor mental health directly affects the treatment outcome of medical illness that in turn increases the use of health care services.^12^ For the patients in our study, this could mean that simply treating the medical injury following self-harm may not be enough if the underlying issue of CSU is also not treated. Research is needed to explore how psychiatric services can be included in primary health care to ensure that patients have access to the necessary help without exacerbating the demands made on limited health care resources.

### Limitations

Data were collected in one ED in an urban city of SA, making it difficult to generalise the findings. Furthermore, data collection relied on self-report measures of CSU. Future studies will be strengthened by including a range of other primary health care settings and including more objective measures of substance use.

## Conclusion

The findings from this study demonstrate that more than a third of the self-harm patients visiting this primary health care setting had a history of CSU. Our study also showed that a smaller proportion of CSU patients received a psychiatric assessment, despite the finding that half of the CSU patients (50.6%) reported a history of self-harm. These findings are particularly concerning in LMICs, like SA, where primary health care facilities are often poorly resourced, understaffed, overcrowded and open for limited hours.^14,15^ A visit to primary health care services that includes a psychiatric assessment could ensure that self-harm patients receive the care that they need, as it provides an opportunity to reinforce health behaviour patterns, to make appropriate referrals and to prevent repetition of self-harm.^22,32^ Despite the benefit of a psychiatric assessment in reducing self-harm, such assessments are not routinely performed before discharge from the ED.^33^ In SA, early detection and necessary care is not always feasible or available to everyone, as limited skills and resources hinder adequate and timeous treatment or referral.^34^ More research is required to determine ways of improving resources available to primary health care services or integrating psychiatric services in primary health care that effectively address CSU and self-harm, as this would seem to be an important component of suicide prevention.

## Footnotes

1 The term ‘mixed race’ may be offensive in some countries; however, this is an official term used in South Africa.
